# Evidence of sequestration of triclabendazole and associated metabolites by extracellular vesicles of *Fasciola hepatica*

**DOI:** 10.1038/s41598-020-69970-4

**Published:** 2020-08-10

**Authors:** Chelsea N. Davis, Ana Winters, Ivana Milic, Andrew Devitt, Alan Cookson, Peter M. Brophy, Russell M. Morphew

**Affiliations:** 1 Institute of Biological, Environmental and Rural Sciences, Aberystwyth University, Aberystwyth, UK; 2 School of Life and Health Sciences, Aston University, Birmingham, UK

**Keywords:** Biochemistry, Biological techniques, Cell biology, Molecular biology, Zoology, Diseases, Medical research, Pathogenesis

## Abstract

Fascioliasis is a neglected zoonotic disease that infects humans and ruminant species worldwide. In the absence of vaccines, control of fascioliasis is primarily via anthelminthic treatment with triclabendazole (TCBZ). Parasitic flatworms, including *Fasciola hepatica,* are active secretors of extracellular vesicles (EVs), but research has not been undertaken investigating EV anthelmintic sequestration. Adult *F. hepatica* were cultured in lethal and sub-lethal doses of TCBZ and its active metabolites, in order to collect EVs and evaluate their morphological characteristics, production and anthelmintic metabolite content. Transmission electron microscopy demonstrated that *F. hepatica* exposed to TCBZ and its metabolites produced EVs of similar morphology, compared to non-TCBZ exposed controls, even though TCBZ dose and/or TCBZ metabolite led to measurable structural changes in the treated *F. hepatica* tegument. qNano particle analysis revealed that *F. hepatica* exposed to TCBZ and its metabolites produced at least five times greater EV concentrations than non-TCBZ controls. A combined mass spectrometry and qNano particle analysis confirmed the presence of TCBZ and the TCBZ–sulphoxide metabolite in anthelmintic exposed EVs, but limited TCBZ sulphone was detectable. This data suggests that EVs released from adult *F. hepatica* have a biological role in the sequestration of TCBZ and additional toxic xenobiotic metabolites.

## Introduction

Fascioliasis is a neglected tropical disease which infects humans and ruminant species globally^1,2^. Millions of people are at risk of this zoonotic disease, with at least 2.4 million people currently infected with fascioliasis in over seventy countries^3^. Worldwide, the disease costs the livestock industry €2.5 billion annually, mainly due to significantly impacting upon animal health and food security^4^. Fascioliasis is controlled by anthelmintic treatment, due to the absence of a commercial vaccine. Triclabendazole (TCBZ) is the anthelminthic treatment primarily used, due to its efficiency against adult and juvenile infections*.* Heavy reliance upon TCBZ in the past 20 years for treating ruminant species and more recently humans, has led to TCBZ resistance, putting treatment strategies at risk^5^.

TCBZ (6-chloro-5(2-3 dichlorophenoxy)-2-methyl thio-benzimidazole) is a halogenated benzimidazole thiol derivative that is distinguished from other benzimidazoles by a chlorinated benzene ring and the absence of a carbamate moiety^6^. Following administration, TCBZ is metabolised in the host liver by sulphoxidation or hydroxylation into TCBZ sulphoxide (TCBZ–SO), TCBZ sulphone (TCBZ–SO_2_), hydroxy-TCBZ (TCBZ-OH), hydroxy-TCBZ–SO (TCBZ–SO-OH) or hydroxy-TCBZ–SO_2_ (TCBZ–SO_2_-OH). TCBZ–SO is reported as the most active metabolite, followed by TCBZ–SO_2_ and TCBZ, where hydroxylated metabolites are less active^7,8^. The TCBZ sulphoxidation metabolism pathway primarily involves the flavin-monooxygenase system, although flavin-monooxygenase and cytochrome P450 enzymatic systems are similarly involved in sulphonation of TCBZ–SO to TCBZ–SO_2_^8,9^. In the host, the timing of peak metabolite plasma levels are known to vary dependent on metabolite, TCBZ–SO 18–24 h, TCBZ–SO_2_ 36–48 h, TCBZ–OH 8 h, TCBZ–SO-OH 21 h and TCBZ–SO_2_-OH 36 h. However, TCBZ–SO_2_ has been detected in plasma up to 5 days post-treatment^7^. TCBZ metabolites strongly bind to plasma proteins, especially albumin, extending their activity in the host. Alternatively, TCBZ metabolites concentrate in the bile ducts^7^. It has been demonstrated that sulpho-reduction and oxidation can be performed by rumen microbes^7,8^. This suggests that gradual TCBZ release from plasma binding and rumen microflora metabolism prolongs exposure in the parasite host environment.

The primary mode for TCBZ metabolite toxicity towards *Fasciola hepatica* is inconclusive since TCBZ metabolites interact with many biological systems and exert cascading effects which have not all been explored^5^. However, as TCBZ has similar biochemistry to benzimidazoles, which bind to the colchicine binding site on β-tubulin molecule receptors, the most expected mode of action involves disruption of microtubule-based processes. In *F. hepatica*, this has been supported by tegumental morphological studies where the adult or juvenile tegument has become synthetically inactive, disrupted and even autophagic when exposed to TCBZ, TCBZ–SO or TCBZ–SO_2_ alongside colchicine microtubule inhibitors and tubulozole microtubule inhibitors as positive controls^9–12^. In addition, loss of tubulin immunoreactivity in the tegumental syncytium, egg formation disruption and ovary, testes and vitelline cell apoptosis has been demonstrated, further supporting that TCBZ disrupts microtubule-based processes in *F. hepatica*^12–15^. Uncertainty upon the TCBZ primary mode of action being disruption of microtubule-based processes, is in part due to the unknown binding site of TCBZ and its metabolites^5,6,11^. Biological mechanisms such as the uncoupling of oxidative phosphorylation^16^, the inhibition of protein synthesis^17^ and the stimulation of glucose derived acetate and propionate^18^ have alternatively been investigated as likely TCBZ modes of action upon *F. hepatica*.

TCBZ metabolites enter *F. hepatica* via oral ingestion or trans-tegumental diffusion; the latter of which is dependent upon the diffusion surface area, TCBZ concentration gradient, environmental acidity and TCBZ lipophilicity^19,20^. Alongside TCBZ, the metabolites TCBZ–SO and TCBZ–SO_2_ have been recovered from *F. hepatica* within 15 min of in vitro incubation, demonstrating rapid diffusion of TCBZ into *F. hepatica*^20^.

In *F. hepatica*, subpopulations of extracellular vesicles (EVs), determined by size and cargo content, have been found to be released from gastrodermal cells and tegumental cells^21,22^. EVs are heterogeneous spherical structures on a nanometre scale, that are limited by a lipid bilayer and contain cytoplasmic components^23^. To clarify, EVs is a collective term covering various subtypes of cell-released membranous structures called exosomes, microvesicles, microparticles, ectosomes, apoptotic bodies and many other names^24^. To the best of the authors knowledge, only one study has been previously undertaken to investigate anti-parasitic drug exposure and parasite EV production^25^. However, in non-parasitic research, the role of EVs during drug exposure is an area of intensive research specifically in the cancer and bacteriology fields. EVs have been observed to incorporate anti-cancer drugs, remove anti-cancer drugs from cancer cells and mediate cell acquired drug resistance^26^. EVs have otherwise been investigated during drug exposure using gram negative and gram-positive bacteria cells^27–29^. The primary reliance upon TCBZ to treat humans and livestock against fascioliasis, where resistance is prevalent, indicates the necessity of further TCBZ pharmacology research upon *F. hepatica*.

This novel study has discovered that *F. hepatica* exposed to TCBZ and its metabolites produced at least five times greater EV concentrations compared to treatment controls. In addition, TCBZ and its metabolites were observed in samples where *F. hepatica* was exposed to TCBZ and TCBZ–SO, although little was identified in samples where *F. hepatica* was exposed to TCBZ–SO_2_. Therefore, these results suggest that EVs are utilised to remove key TCBZ metabolites from the parasite’s microenvironment, to sustain *F. hepatica* survival.

## Methods

### Fasciola *hepatica* culture and EV collection

Adult *F. hepatica *in vitro culture was revised from Morphew et al.^30^. Adult *F. hepatica* (n = 420) were retrieved from naturally infected ovine livers immediately post-slaughter from a local abattoir in mid Wales, U.K, during two visits. Immediately after collection, *F. hepatica* were thoroughly washed in phosphate buffered saline (PBS), pH 7.4, at 37 °C to remove host material. Replicates of ten adult similar sized matched *F. hepatica* were then cultured in 30 ml (3 ml/*F. hepatica*) culture media (Dulbecco’s modified Eagle’s medium (DMEM) (w/o NaHPO_3_ and PO_4_) plus 2.2 mM Ca (C_2_H_3_O_2_), 2.7 mM MgSO_4_, 61.1 mM glucose, 1 μM serotonin, 5 μg/ml gentamycin, 15 mM N-2-hydroxyethylpiperazine-N′-2-ethanesulfonic acid (HEPES), pH 7.4) in a falcon tube (Fisher Scientific) at 37 °C for 4 h, including transport to the laboratory, to establish a *F. hepatica* biological baseline (baseline controls). For control samples, 30 ml culture media in a falcon tube was similarly incubated at 37 °C for 4 h. Identical experimental procedures were undertaken after each abattoir visit.

Following initial culture, media was replaced and supplemented with either TCBZ (Sigma), TCBZ–SO (Sigma) or TCBZ–SO_2_ (Sigma) at 50 μg/ml (lethal dose) or 15 μg/ml (sub-lethal dose) in dimethyl sulfoxide (DMSO) (Sigma) (final conc. 0.1%, v/v) (anthelmintic exposure samples)^31^. Combining experiments undertaken after each abattoir visit, in total 60 *F. hepatica* were used for each TCBZ metabolite and concentration treatment, which were separated into six falcon tubes (ten *F. hepatica* per falcon tube) (n = 6). Therefore, in total for anthelmintic treatment samples, 360 *F. hepatica* were used. All samples were incubated at 37 °C for 5 h, before *F. hepatica* were either snap frozen in liquid nitrogen or stored in ethanol for transmission electron microscopy analysis. Culture media was frozen and stored at − 80 °C until further use.

Three control groups were used in the study: treatment control, anthelmintic control and EV control. After initial culture, combining experiments undertaken after each abattoir visit, in total; the treatment controls included 60 *F. hepatica* separated into six falcon tubes (ten *F. hepatica* per falcon tube) containing 30 ml culture media and DMSO (final conc. 0.1%, v/v) (n = 6); the EV controls included three falcon tubes containing 30 ml culture media, DMSO (final conc. 0.1%, v/v) and size exclusion chromatography (SEC) purified EVs previously derived from ten *F. hepatica* (the falcon tubes did not contain *F. hepatica)* (n = 3); the anthelmintic controls included three falcon tubes either containing TCBZ (n = 3), TCBZ–SO (n = 3) or TCBZ–SO_2_ (n = 3) at 50 μg/ml (lethal dose) in 30 ml culture media and DMSO (final conc. 0.1%, v/v) (the falcon tubes did not contain *F. hepatica* or EVs). All control samples were incubated at 37 °C for 5 h, before the media was frozen and stored at − 80 °C until further use.

The viability of *F. hepatica* were scored with reference to Morphew et al*.*^30^ when collected at the abattoir (zero hour), after pre-incubation washing (cumulative 1 h), after 4 h baseline in vitro maintenance (cumulative 5 h) and after 5 h TCBZ/SO/SO_2_ exposure in vitro maintenance (cumulative 10 h) (supplementary data S1). *F. hepatica* were classed as viable if they had a score > 1, a score of 1 deemed reduced viability and a score of 0 denoted non-viable *F. hepatica*.

### EV purification using size exclusion chromatography

*Fasciola hepatica* EV purification was followed from Davis et al*.*^32^. Briefly, culture media was centrifuged at 300×*g* for 10 min at 4 °C and then at 700×*g* for 30 min at 4 °C, removing any large particulates. The samples were then concentrated using 10 KDa MWCO Amicon ultra-15 centrifugal filter units (Merck Millipore), following the manufacturer’s guidelines. Briefly, the samples were centrifuged at 4000×*g* for 20 min at 4 °C, until approximately 500 µl of samples were retained in the filter. The samples were passed through qEVoriginal SEC columns (IZON science), utilising the manufacturer’s optimised protocol. Briefly, the column was rinsed with 10 ml of filtered (0.2 μm, syringe filter, Life Sciences) PBS. The samples were then added to the SEC column and the first 2.5 ml of flow through was discarded. The next 2.5 ml of flow through, containing EVs, was collected and stored at − 80 °C for further analysis.

### Transmission electron microscopy (TEM)

*Fasciola hepatica* specimens from in vitro culture, were fixed in 2.5% v/v glutaraldehyde in 0.1 M sodium cacodylate (Agar Scientific), pH 7.2, overnight at 4 °C. Samples were then washed twice in wash buffer (0.1 M sodium cacodylate, pH 7.2) and then placed in wash buffer containing 1% w/v osmium tetroxide solution (Agar Scientific) for 1 h. Samples were drained and two further buffer washes containing sodium cacodylate only were undertaken. Samples were then placed in an ultra-pure water bath and dehydrated in a series of ethanol dilutions (30%, 50%, 70%, 95% and 100% v/v) for 1 h. Samples were embedded in resin, whereby infiltration was achieved using ethanol mixtures (2:1, 1:1 and 1:2) and LR white (hard grade) resin (London Resin Company Ltd). Samples were transferred to gelatine or polyethylene moulds and polymerised overnight at 60 °C. Sections (1–2 μm) were cut and dried onto glass slides, before staining with azur II (Sigma) or methylene blue (Sigma) for 2 min. Ultrathin (60–80 nm) sections were then cut using Reichert-Jung ultracut E ultramicrotome with a diatome ultra 45° diamond knife and collected on nickel slot grids (GS2X0.5 3.05 mm diameter gilder grids, Grantham, UK) float-coated with butvar B98 polymer films (Agar Scientific). All sections were double-stained with a 4:1 dilution of 5% w/v uranyl acetate (Agar Scientific) in iso-propyl alcohol (Sigma) and Reynold's lead citrate (6.65 g of lead nitrate mixed with 8.80 g of tri-sodium citrate in 100 ml distilled water) (TAAB Laboratories Equipment Ltd, Aldermaston, UK) for tegument TEM imaging (Jeol JEM1010 microscope at 60 kV).

*Fasciola hepatica* EV TEM methodology was followed from Davis et al.^32^. Briefly, EV samples were fixed onto formvar/carbon coated copper grids (Agar Scientific) by adding 10 µl sample to the grid for 45 min on ice. Grids were then placed on the viscous of 4% v/v uranyl acetate for 5 min on ice. Grids were stored at room temperature for at least 24 h before being imaged using the TEM (Jeol JEM1010 microscope at 60 kV).

Images were photographed on Carestream 4489 electron microscope film (Agar Scientific). Film images were developed (Kodak D-19 developer) for 4 min at 20 °C, before being fixed, washed and dried according to the manufacturer's instructions. The resulting negatives were scanned (Epson Perfection V800 film scanner) and converted to positive images. The size of 100 EVs from anthelmintic exposure samples and treatment controls were measured (nm) using ImageJ (https://imagej.nih.gov/ij/) as described previously^32^.

### Particle size and concentration quantification by qNano particle analysis

EV samples or calibration particles (CPC200, 1:1000 filtered (0.2 μm, Life Sciences) PBS dilution, Izon science) were placed in the Nanopore (NP200, Izon science) on the qNano device (Izon science). All samples and calibration particles were measured at 47 mm nanopore stretch with a voltage of 100 nA at pressure level 7 mbar. Short current pulses detected the particles. Sample analysis was conducted using qNano particulate analysis (Izon, version 3.2).

### EV sample preparation, mass spectrometry and TCBZ/SO/SO_2_ identification

One millilitre of all TCBZ/SO/SO_2_ exposure EV samples and control samples was ultra-centrifuged (S55-S rotor, Sorval MX120 centrifuge, Thermo scientific) at 100,000×*g* for 30 min at 4 °C and the PBS supernatant was removed. Next, 500 μl Absolute ethanol (VWR) was added to the pellet, before sonicating for 30 s and resting on ice for 30 s. This was repeated twice to lyse EVs. Ethanol was then evaporated, using a vacuum concentrator (Maxi dry plus, Heto) and 100 μl 70% methanol (VWR) was added to the pellet. The samples were vortexed for 30 min, before centrifuging at 10,000×*g* for 2 min. TCBZ/SO/SO_2_ metabolite standard samples were prepared using concentrations: 100 ng/ml, 75 ng/ml, 50 ng/ml, 25 ng/ml, 10 ng/ml, 5 ng/ml and 2.5 ng/ml in 100 μl 70% methanol and were vortexed and centrifuged at 10,000×*g* for 2 min. 20 μl of all sample supernatants and standard supernatants were then prepared in sampling vials (Thermo scientific) and randomly ordered for mass spectrometry.

Metabolites were evaluated as described in Akpanika et al.^33^ using reverse-phase high performance liquid chromatography with online photodiode array detection and electrospray ionisation-ion trap tandem mass spectrometry (HPLC–PDA-ESI/MSn). Structural elucidation was completed on a Thermo Finnigan liquid chromatography mass spectrometry system (Thermo Electron Corporation) including a Finnigan photo diode array plus detector, a Finnigan linear trap quadrupole with electrospray ionization source, and a C18 Nova-Pak column (3.9 × 100 mm, particle size 4 μm, Waters) where the column oven temperature was maintained at 30 °C. The photodiode array detection scan range was 240–400 nm, with 10 μl injection volume. The mobile phase included water with 0.1% v/v formic acid (solvent A) and methanol with 0.1% v/v formic acid (solvent B). The column was equilibrated using 95% v/v solvent A with a 1 ml min^−1^ flow rate, where 10% entered the mass spectrometer, and solvent B percentage increased linearly to 65% v/v over 60 min. Mass spectrometry parameters were: sheath gas 30, auxiliary gas 15, sweep gas zero (all arbitrary units), spray voltage − 4.0 kV in negative and 4.8 kV in positive ionisation mode, capillary temperature 320 °C, capillary voltage − 1.0 V and 45.0 V respectively, tube lens voltage − 68 V and 110 V respectively, and normalised collision energy 35%.

Mass spectrometry spectrum peaks for anthelmintics: TCBZ, TCBZ–SO, TCBZ–SO_2,_ TCBZ-OH, TCBZ–SO-OH and TCBZ–SO_2_-OH in all TCBZ/SO/SO_2_ exposure samples, TCBZ metabolite standard samples and control samples were manually identified by molecular weight and scan time using Xcalibur software (Version 3.0) (Thermo scientific) (supplementary data S2). The spectrum peak area of all samples was recorded. TCBZ/SO/SO_2_ metabolite standard sample spectrum peak areas were used to create standard curves for TCBZ/SO/SO_2_ metabolite quantification (ng) of TCBZ/SO/SO_2_ exposure samples and control samples. The amount of TCBZ/SO/SO_2_ metabolite found in EVs (pg/particle^9^) was determined by spectrum peak area quantification (ng) and qNano particle analysis sample concentration (particle/ml) in all TCBZ/SO/SO_2_ exposure samples and control samples. Any background metabolite concentration found in TCBZ/SO/SO_2_ control samples, was deducted from TCBZ/SO/SO_2_ exposure samples and other control samples. Sample spectrum peak areas below 1000 were disregarded from the results, as this data could not be confidently defined from spectra background noise.

### Statistical analysis

The Shapiro–Wilk test and quantile–quantile plots were used to identify data normality (*p* > 0.05). Kruskal–Wallis one-way analysis of variance with post hoc Nemenyi test was used to analyse EV diameter measurements using TEM and ImageJ analysis. The independent welch two samples T-test, a one-way or a two-way ANOVA with post-hoc Fisher’s least significant difference test was conducted to identify significant differences between TCBZ/SO/SO_2_ exposure samples and control samples using qNano particle analysis and mass spectrometry. One outlier from the treatment controls and lethal TCBZ–SO_2_ exposure sample was omitted in the particle diameter data. In addition, one sub-lethal and lethal TCBZ–SO exposure replicate was omitted prior to statistical analysis in Fig. 5, as the sample spectrum peak areas were below 1000.

## Results

### Fasciola *hepatica* survival

All *F. hepatica* survived 10 h in vitro maintenance, where only 2% *F. hepatica* sub-lethal TCBZ/SO/SO_2_ and lethal TCBZ–SO exposure samples, 3% *F. hepatica* lethal TCBZ exposure samples, and 7% *F. hepatica* lethal TCBZ–SO_2_ exposure samples presented reduced viability (viability score of 1) (supplementary data S1).

### EV morphological characteristics comparison

**Figure 1:**
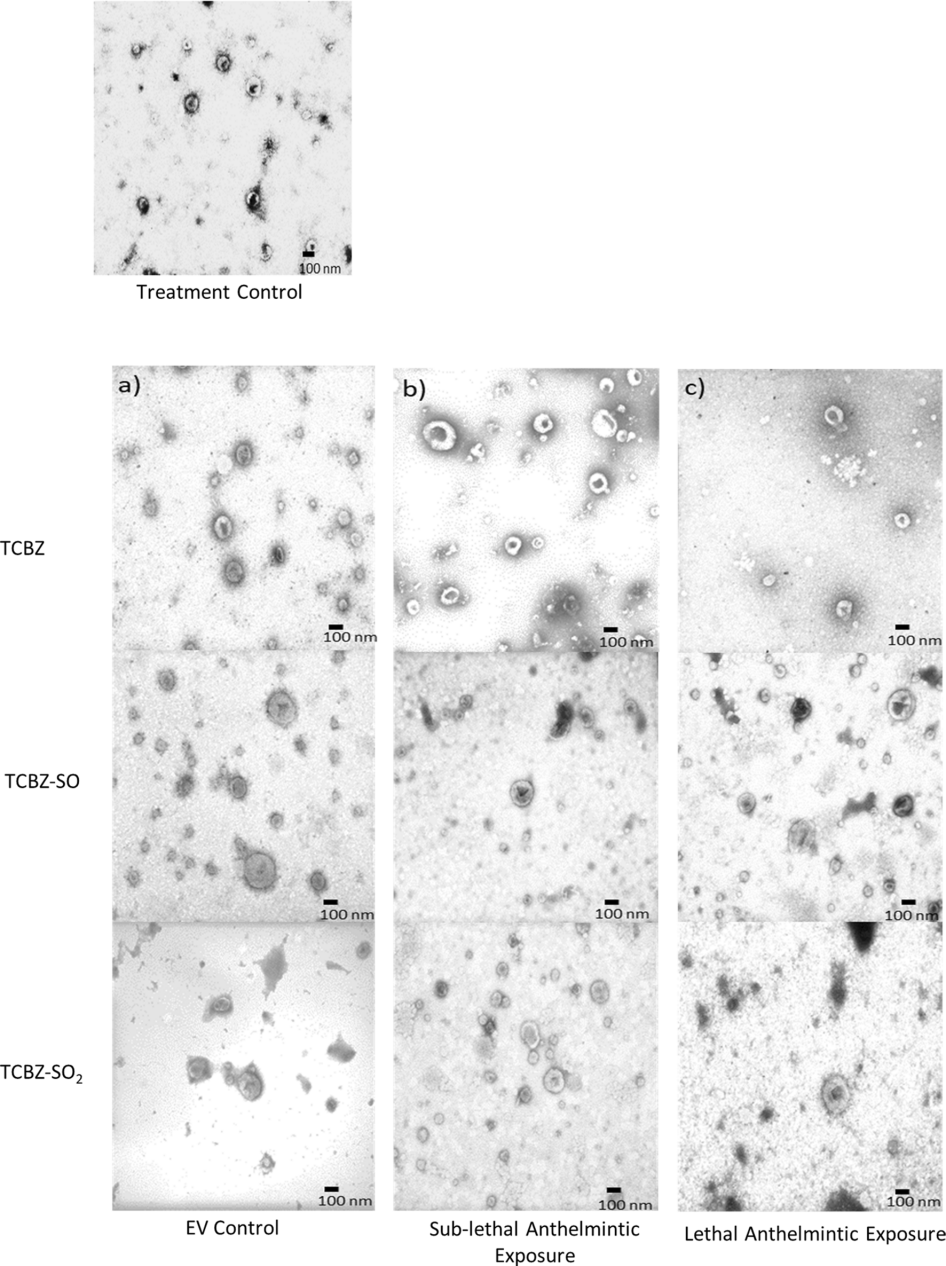
TEM micrographs demonstrating *F. hepatica* EVs from the treatment control (n = 3) and **a** SEC purified EVs (EV control) dosed with lethal (50 μg/ml) TCBZ, TCBZ–SO or TCBZ–SO_2_ in DMSO (0.1%, v/v) from top to bottom, respectively (n = 3), **b** EVs from adults *F. hepatica* exposed to sub-lethal (15 μg/ml) doses of TCBZ, TCBZ–SO or TCBZ–SO_2_ in DMSO (0.1%, v/v) from top to bottom, respectively (n = 3) and **c** EVs from adults *F. hepatica* exposed to lethal (50 μg/ml) doses of TCBZ, TCBZ–SO or TCBZ–SO_2_ in DMSO (0.1%, v/v) from top to bottom, respectively (n = 3). Images were captured at 50× magnification.

**Figure 2:**
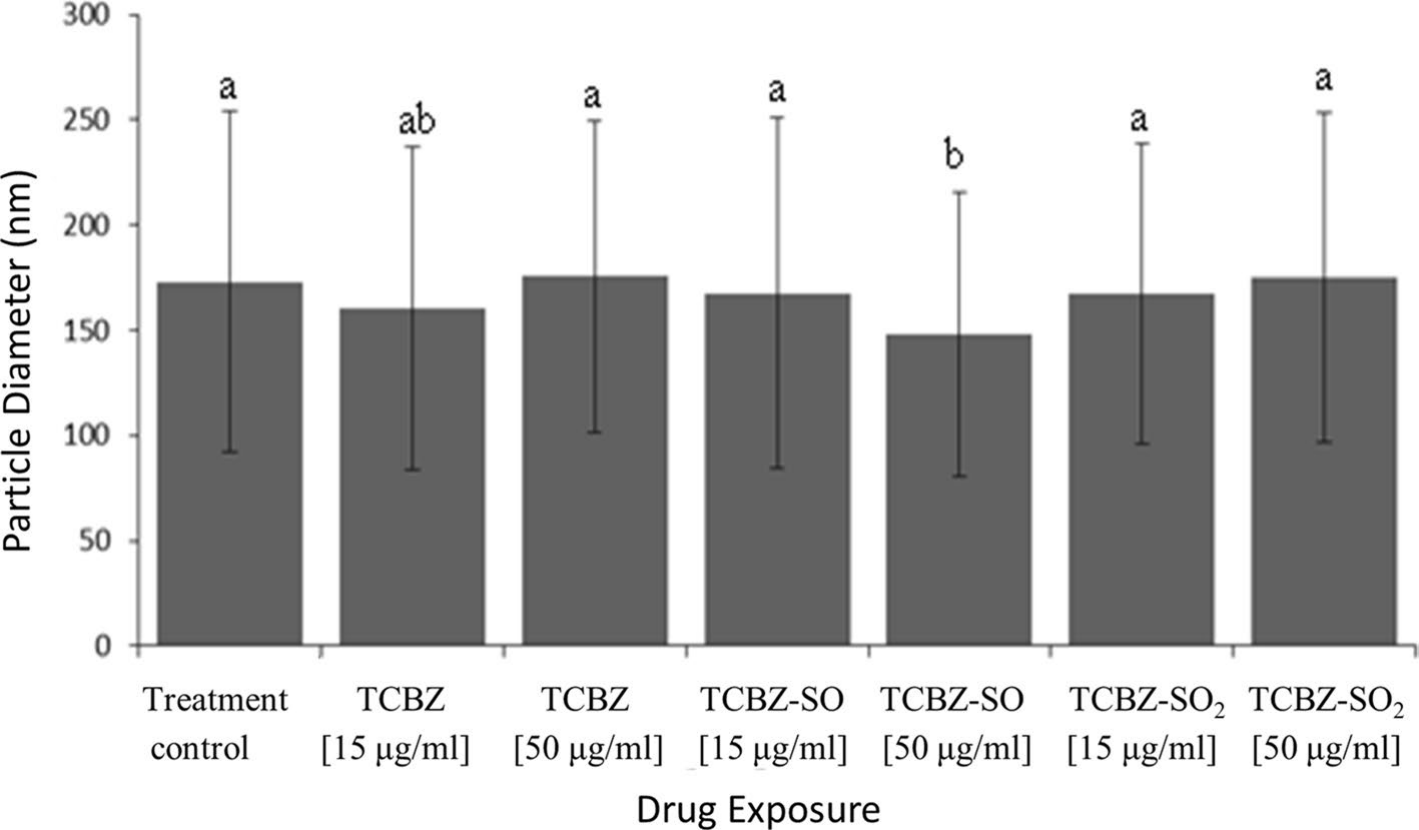
Particle diameter (nm) of control (n = 6) or drug exposure samples with lethal (50 μg/ml) or sub-lethal (15 μg/ml) doses of TCBZ/SO/SO_2_ (weighted mean ± weighted SD) (n = 6), where letters a and b represent significance (*p* < 0.05). One-way ANOVA and Fisher’s LSD identified significance (*p* < 0.05) between drug exposure samples and the treatment control (*F*_(6,34)_ = 2.7, *p* = 0.03). Two way ANOVA identified no significance between particle diameter and lethal or sub-lethal drug dose (F_(2,1)_ = 0.1, *p* = 0.80), as well as drug metabolite (F_(2,1)_ = 2.8, *p* = 0.08). One outlier was removed from lethal TCBZ–SO_2_ drug dose (n = 5).

TEM micrographs identified that lethal or sub-lethal TCBZ/SO/SO_2_ exposure samples had similar EV morphology, compared to treatment and EV control samples (Fig. 1). When using qNano particle analysis, particle diameter was similar between all TCBZ/SO/SO_2_ exposure samples and the treatment controls (TCBZ [15 μg/ml] = 160.4 nm ± 77.1 SD, TCBZ [50 μg/m] = 175.5 nm ± 74.2 SD, TCBZ–SO [15 μg/ml] = 167.6 nm ± 83.5 SD, TCBZ–SO_2_ [15 μg/ml] = 167.6 nm ± 71.5 SD, TCBZ–SO_2_ [50 μg/ml] = 177.2 nm ± 78.4 SD, treatment control = 173.1 nm ± 81.3 SD), with the exception of the lethal TCBZ–SO exposure samples (TCBZ–SO [50 μg/ml] = 147.9 nm ± 67.4 SD), which had significantly smaller particle diameter size compared to the treatment controls (F_(6,34)_ = 2.7, *p* = 0.03) (Fig. 2). Two way ANOVA identified that there was no significance between particle diameter and lethal or sub-lethal anthelmintic dose (F_(1,31)_ = 0.0, *p* = 0.94), as well as TCBZ metabolite (F_(2,31)_ = 2.3, *p* = 0.11).

Kruskal–Wallis one-way analysis of variance showed there was a significant statistical difference (χ^2^_(6)_ = 56.96, *p* < 0.001) between treatment and control sample EV diameter using TEM image analysis, where lethal TCBZ (TCBZ [50 μg/m] = 53.2 nm ± 44.1 SD, *p* = 0.01) and TCBZ–SO (TCBZ–SO [50 μg/m] = 48.8 nm ± 29.9 SD, *p* = 0.009) exposure samples had significantly smaller diameter size compared to the treatment controls (treatment control = 62.6 nm ± 32.4 SD). In addition, sub-lethal TCBZ exposure samples (TCBZ [15 μg/ml] = 74.3 nm ± 33.8 SD, *p* < 0.001) were observed to have a significantly higher diameter size compared to anthelmintic exposure samples (TCBZ–SO [15 μg/ml] = 61.9 nm ± 51.2 SD, TCBZ–SO_2_ [15 μg/ml] = 52.6 nm ± 29.2 SD, TCBZ–SO_2_ [50 μg/ml] = 51.6 nm ± 31.3 SD).

### EV release comparison

**Figure 3:**
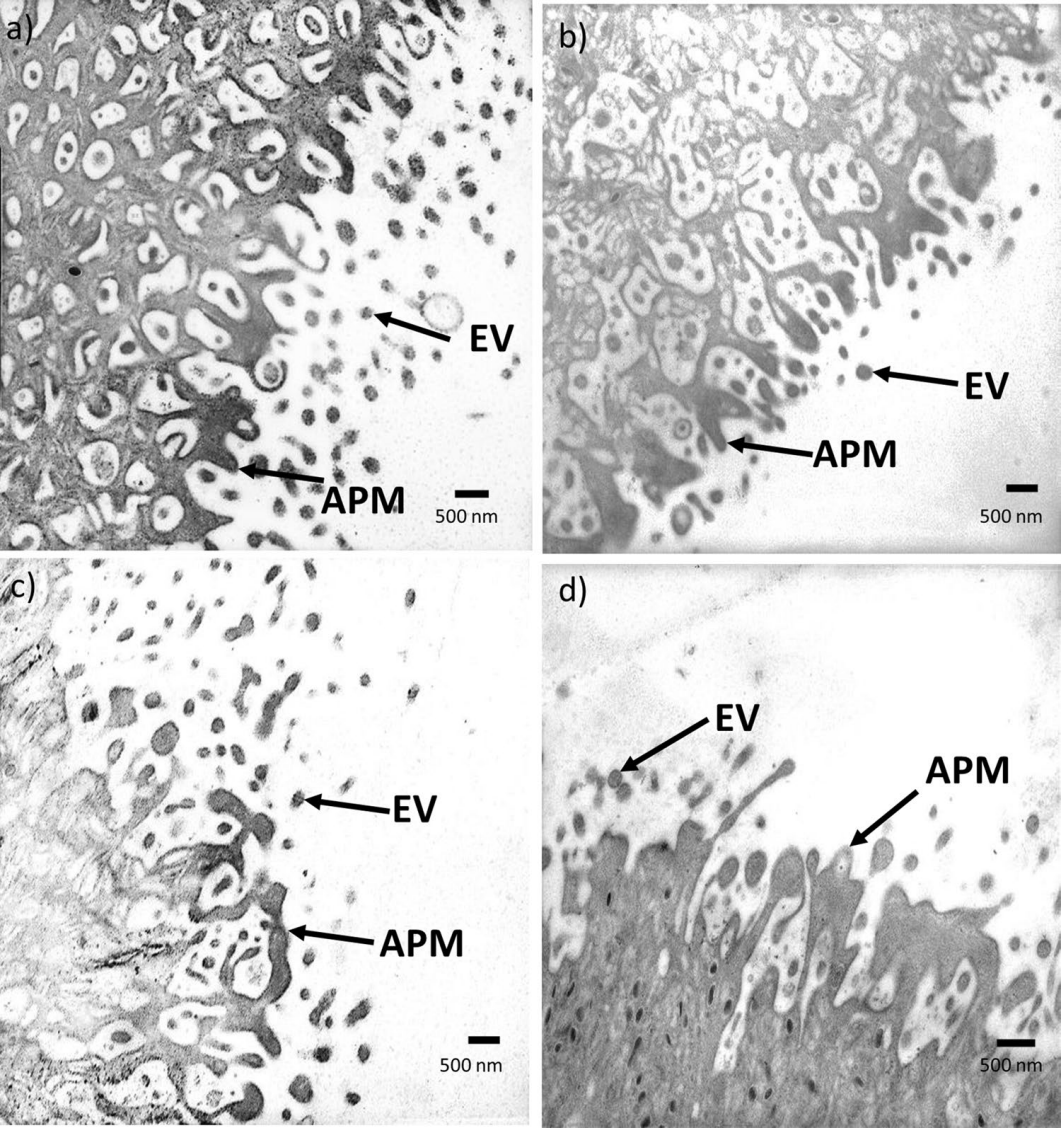
TEM micrographs showing the tegument of *F. hepatica* anthelmintic exposure and treatment control samples where **a** DMSO (0.1%, v/v) only (treatment control) (n = 3), **b** TCBZ (50 μg/ml) in DMSO (0.1%, v/v) (n = 3), **c** TCBZ–SO (50 μg/ml) in DMSO (0.1%, v/v) (n = 3), or d) TCBZ–SO_2_ (50 μg/ml) in DMSO (0.1%, v/v) (n = 3). Images were taken at 15× magnification and arrows indicate tegument structural changes such as disorganised tissues and small spaces in the tissue at the apical plasma membrane (APM), and EV release (EV) following anthelmintic exposure, compared to the treatment control.

TEM micrographs demonstrated that EV release was similar in all TCBZ/SO/SO exposure samples, compared to the treatment controls (Fig. 3). Although, the tegument showed clear structural changes where disorganised tissues and small spaces in the tissue were noted following anthelmintic exposure, especially when exposed to TCBZ–SO, compared to the treatment controls.

**Figure 4:**
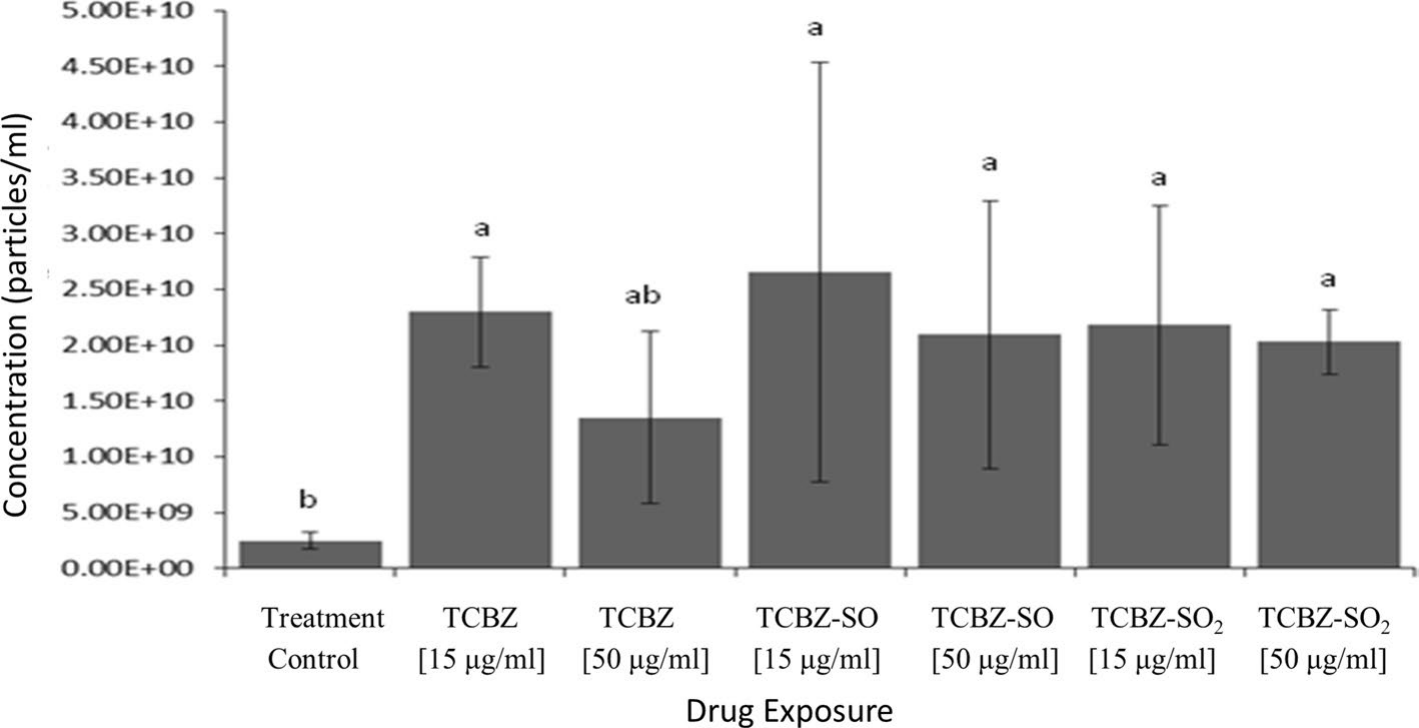
Particle concentration (particles/ml) of control (n = 5) or anthelmintic exposure (n = 6) with lethal (50 μg/ml) or sub-lethal (15 μg/ml) doses of TCBZ, TCBZ–SO or TCBZ–SO_2_ (mean + SD) where letters a and b represent significance (*p* < 0.05). One-way ANOVA and Fisher’s LSD test identified significance (*p* < 0.05) between drug exposure samples and the treatment control (F(6,34) = 2.7, *p* = 0.03). Two way ANOVA identified that there was no significance between particle diameter and lethal or sub-lethal drug dose (F(1,32) = 2.0, *p* = 0.17), as well as anthelmintic metabolite (F(2,32) = 0.7, *p* = 0.53). One outlier was removed from the control (n = 5).

qNano particle analysis demonstrated that TCBZ/SO/SO_2_ exposure samples had at least five times greater particle concentration than the treatment controls (Fig. 4). Specifically, there was a significantly greater particle concentration between sub-lethal TCBZ dose, lethal and sub-lethal TCBZ–SO, lethal and sub-lethal TCBZ–SO_2_ dose exposure compared to the treatment controls (F_(6,34)_ = 2.7, *p* = 0.03). Two way ANOVA identified that there was no significance between particle concentration and lethal or sub-lethal anthelmintic dose (F_(1,32)_ = 2.0, *p* = 0.17), as well as anthelmintic metabolite (F_(2,32)_ = 0.7, *p* = 0.53).

### EV TCBZ/SO/SO_2_ uptake comparison

**Figure 5:**
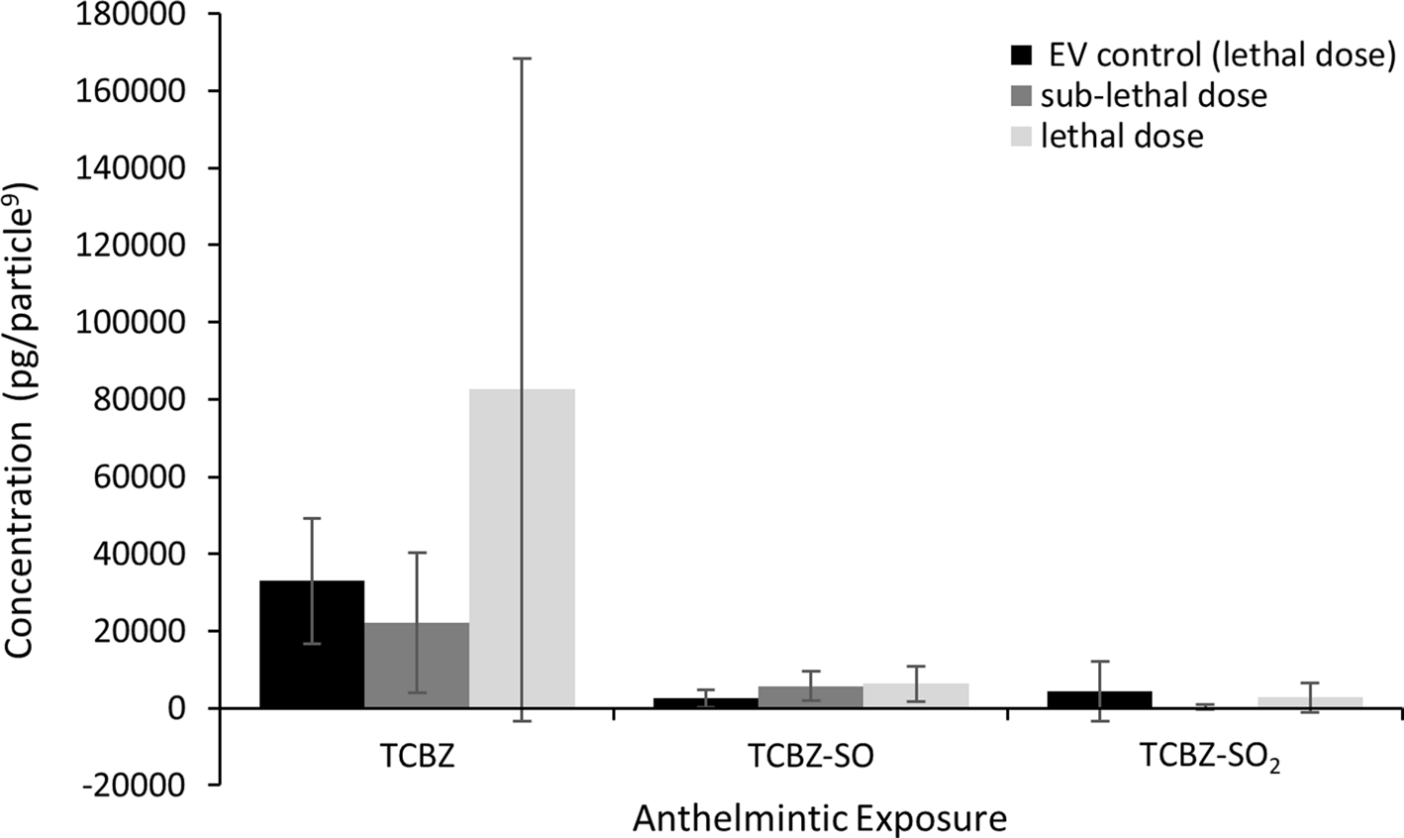
EV drug concentration (pg/particle^9^) in EV control (50 ug/ml) (n = 3) or drug exposure samples with lethal (50 μg/ml) or sub-lethal (15 μg/ml) doses of TCBZ (n = 6), TCBZ–SO (n = 5) or TCBZ–SO_2_ (n = 5) (mean ± SD). Two way ANOVA identified that there was no significance between lethal and sub-lethal EV anthelmintic concentration (F_(1,19)_ = 2.7, *p* = 0.12) between TCBZ and TCBZ–SO exposure treatments but there was a significantly greater TCBZ concentration in EVs (n = 6) in comparison to TCBZ–SO EV anthelmintic concentrations (n = 5) (F_(1,19)_ = 5.1, *p* = 0.04). One TCBZ–SO sub-lethal and lethal anthelmintic exposure replicate was omitted from statistical analysis, as sample spectrum peak areas were below 1000.

No anthelmintic metabolites were identified in the baseline controls and treatment controls, confiming that there was no cross-contamination of anthelmintic, while undertaking methodology protocols (supplementary data S2). Anthelmintic metabolites were observed in two TCBZ–SO and TCBZ–SO_2_ anthelmintic control samples, so metabolite concentations were averaged and deducted from TCBZSO/SO_2_ EV controls and TCBZSO/SO_2_ exposure sample results, to remove the maximum TCBZSO/SO_2_ concentration background which was possibly not uptaken by EVs but still remained in samples. One sub-lethal and lethal TCBZ–SO sample and one sub-lethal and lethal TCBZ–SO_2_ sample were observed to have a spectrum peak area below 1000, and thus were removed from the results, as samples could not be confidently defined from spectra background noise. Anthelmintic concentrations in EVs were observed in all TCBZ exposed samples and all TCBZ–SO exposed samples, other than one TCBZ–SO EV control replicate. TCBZ–SO_2_ concentrations were observed in only one replicate of TCBZ–SO_2_ EV control, one replicate of TCBZ–SO_2_ sub-lethal exposure sample and two replicates of lethal TCBZ–SO_2_ exposure samples (Fig. 5). There was a significantly greater TCBZ concentration in EVs when *F. hepatica* was exposed to TCBZ (n = 6), in comparison to TCBZ–SO anthelmintic concentrations in EVs when *F. hepatica* were exposed to TCBZ–SO (n = 5) (F_(1,19)_ = 5.1, *p* = 0.04). There was no significance difference between anthelmintic dosage within TCBZ and TCBZ–SO exposure treatment (F_(1,19)_ = 2.7, *p* = 0.12).

TCBZ was identified in two TCBZ–SO EV controls, two lethal TCBZ–SO exposure replicates and one sub-lethal TCBZ–SO exposure replicate. Otherwise, no other alternative anthelmintic metabolites were observed in controls and TCBZ/SO/SO_2_ exposure samples.

## Discussion

Reliance upon TCBZ to treat humans and livestock against fascioliasis, where *F. hepatica* TCBZ resistance has been reported, indicates the urgency to advance TCBZ pharmacology research involving *F. hepatica*. Thus, given the characteristics of extensively documented cancer drug-human EV interactions^26^, it seems logical to investigate the role of *F. hepatica* EVs following adult *Fasciola hepatica* exposure to TCBZ and its metabolites. This research has provided new insights upon how *F. hepatica* EVs are involved in *F. hepatica* survival during anthelmintic exposure, by investigating EV morphological characterisation, EV release and EV anthelmintic metabolite contents.

*Fasciola hepatica* cultured in lethal and sub-lethal doses of TCBZ/SO/SO_2_ had similar EV morphology to treatment control samples and *F. hepatica* EV morphology reported in previous investigations^32^. EV diameter measurements using qNano particle analysis and TEM showed that lethal and sub-lethal doses of TCBZ/SO/SO_2_ were similar sizes to treatment control EV samples, other than *F. hepatica* cultured in lethal TCBZ–SO which had significantly lower particle diameter size. TCBZ–SO is the most active TCBZ metabolite against *F. hepatica*, so it could be possible that the change in EV size, a reduction in this case, is due to metabolite potency^7,8^. Additionally, TEM EV diameter measurements identified that lethal TCBZ had significantly lower particle diameter size compared to treatment control samples and sub-lethal TCBZ exposure samples had significantly higher diameter size compared to anthelmintic exposure samples. Not only were these significant results different between methodologies, but TEM EV diameters were smaller than qNano particle analysis measurements. Comparison between TEM EV diameter measurements and nanoparticle tracking analysis (NTA) similarly found that EVs were smaller using TEM methodology, which was suggested to be due to NTA EV aggregate measurements^34^. Additionally, TEM fixing and staining methodology, which is not required for qNano particle analysis, leads to a characteristic central depression in EV morphology^35^, which is likely to adversely affect EV size and shape. In this case, this outcome is unrelated to *F. hepatica* survival under the conditions and duration of the assays, as lethal TCBZ–SO, lethal TCBZ and sub-lethal TCBZ anthelmintic exposure parasites had similar viability scores as sub-lethal TCBZ/SO/SO_2_ exposure parasites.

Comparable EV release from the tegument was identified when *F. hepatica* were exposed to lethal TCBZ/SO/SO_2_, in comparison to the treatment controls. However, the tegument exhibited structural changes after TCBZ/SO/SO_2_ exposure, especially when exposed to TCBZ–SO metabolite, compared to the treatment controls. Further detailed TEM images of *F. hepatica* tegumental syncytium after 24-h sub-lethal TCBZ/SO/SO_2_ exposure, identified swollen basal infolds and disorganised tissues with small spaces between intact muscle^9^. Anthelmintic exposure also caused reduced secretory bodies, swollen mitochondria and a lack of Golgi complexes in tegumental tissue^9^. Severity of damage largely varied between TCBZ and the two metabolites^9^, as noted in the current study.

*Fasciola hepatica* exposed to lethal and sub-lethal TCBZ/SO/SO_2_ had five-fold greater EV release, compared to the treatment controls. Specifically, there was a significantly greater particle concentration between all sub-lethal anthelmintic exposure samples and lethal TCBZ–SO and TCBZ–SO_2_ exposure samples, compared to the treatment controls. In contrast, the only EV-drug parasite experiment previously undertaken, demonstrated a rapid inhibition of EV release when *Brugia malayi* and *Dirofilaria immitis* were cultured with ivermectin over a 24-h period^25^*.* However, antibiotic treatments upon *S. maltophilia* and *Escherichia coli* cultures have led to increased outer membrane vesicle release^28,29^. Furthermore, photodynamic treatment and chemotherapy increased levels of cancer cell circulating EVs several fold in a dose dependant manner^36^, supporting the current study findings. Other investigations in the cancer field have observed chemotherapeutic treatments inhibiting EV release, temozolomide and geldanamycin glioblastoma treatment decreased microvesicle release dose-dependently, attributing to drug-induced cell death^37^ and exosome concentration decreased two-fold in the urine of prostate cancer patients, following a 12-week treatment of androgen deprivation therapy^38^.

Anthelmintic dose or metabolite did not affect particle concentration, when *F. hepatica* were exposed to lethal and sub-lethal TCBZ/SO/SO_2_. It could be possible that greater EV release is due to anthelmintic toxicity causing tissue disorganisation and spaces between muscles in the tegument syncytium, allowing uncontrolled stress-induced EV release. This proposal would explain why neither the anthelmintic dose used, nor the anthelmintic metabolite chosen, did not specifically influence *F. hepatica* EV release. Interestingly, hepatocellular carcinoma cell exosome secretion was increased by anticancer chemotherapeutics or via heat shock. In this instance, exosomes carried a greater amount of heat shock proteins when exposed to anticancer chemotherapeutics, suggesting exosome release was stress-induced^39^.

TCBZ and TCBZ–SO concentrations were identified in EVs, where *F. hepatica* exposed to TCBZ had a significantly higher TCBZ concentration in EVs, compared to TCBZ–SO. Limited TCBZ–SO_2_ was detected in EVs from *F. hepatica* exposed to TCBZ–SO_2_. EV chemotherapeutic uptake has been recognised in many cancer cell line EV studies^40–47^. As anthelmintic dose did not affect anthelmintic concentration observed in EVs, it is suggested that anthelmintic EV uptake was mostly passive. TCBZ (log Pow = 5.3) is the most hydrophobic metabolite, so is more likely to be distributed in EVs than the other metabolites, while TCBZ–SO_2_ (log Pow = 3.4) is the least hydrophobic metabolite and is less likely to be distributed in EVs compared with other TCBZ metabolites, supporting the current study findings. Similar results were discovered when shed vesicles were isolated from cancer cells which passively accumulated and retained doxorubicin dose-dependently. Thermodynamic binding interactions were suggested to explain chemotherapeutic accumulation, and lipid complex formation was found to be involved in chemotherapeutic retention^48^. A range of methods are used to purposely load treatments into EVs including incubation, where temperatures of 22–37 °C lead to chemotherapeutic EV uptake within a few hours^49^. This indicates that the incubation period for experimental *F. hepatica* culture, and in vivo conditions, may be optimal for anthelmintic passive diffusion into EVs. However, the potential that TCBZ and its metabolites could be up taken by active processes by *F. hepatica* EVs should not be dismissed. Drug accumulation in cell membranes where EVs are produced has been reported to allow drug removal from cancer cells^44,48^. In depth investigations on the EV uptake mechanism of anthelmintics, would be beneficial to improve fascioliasis control strategies.

Alternative TCBZ metabolites were noted in two TCBZ–SO EV controls, two lethal TCBZ–SO and one sub-lethal TCBZ–SO replicates. Sulpho-reduction and oxidation has only been previously noted in sheep ruminal fluid under anaerobic conditions^8^. Phase I detoxification of chemical stressors in adult parasitic helminths is dominated by reductive and hydrolytic-based metabolisms, where sulphoxidation occurs by enzymes in different cellular locations, and TCBZ–SO is oxidised to the less active TCBZ–SO_2_^50,51^. Phase II studies have been focused on glutathione conjugation, with extensive investigations upon the role of glutathione transferase detoxifying reactive oxygen intermediates^50^. Fatty acid binding proteins, in addition to their transport roles for fatty acids, sterols, lysophospholipids and nonpolar organic ions (e.g. haem and bile pigments), also sequester toxins such as reactive oxygen intermediates, so are considered to have evolved phase III detoxification roles^52^. Interestingly, anthelmintic metabolising, sequestration and efflux pump proteins have been observed in the proteomic profile of *F. hepatica* EVs including aldo/keto reductases, amidase, phosphodiesterase, glutathione transferase, methyl transferase, ABC transporter transmembrane, P-glycoprotein and fatty acid binding protein^22,32,53,54^ further suggesting that EVs are organelles with specialised detoxification functions.

In summary, *F. hepatica* exposed to TCBZ and its metabolites produced at least five times greater EV concentrations than controls, albeit this may not be a pro-active and direct parasite detoxification strategy, but indirect via tegumental tissue disorganisation initiated by anthelmintic toxicity. However, the presence of active anthelmintic metabolites were measurable in EVs released from *F. hepatica*. Therefore, anthelmintic uptake by EVs is likely to reduce the availability of at least some active TCBZ compounds in the parasite’s microenvironment, contributing towards *F. hepatica* survival. Additional investigation upon the retention of anthelmintics with in EVs and bespoke mechanisms of EVs to potentially remove or metabolise anthelmintics, would improve our understanding of the significance of our observations. Further research with defined isolates should now be undertaken to determine if there is a contribution provided by *F. hepatica* EVs towards TCBZ resistance mechanisms.

## Supplementary information

Supplementary data S1. (**a**) *F. hepatica* viability scores and their description for the assessment of in vitro anthelmintic exposure^55^ and (*b*) viability of *F. hepatica* (n = 420) survival after 10 h of in vitro maintenance, where culture samples had been subjected to DMSO (0.1%, v/v) only (treatment control) (n = 6) or either TCBZ, TCBZ-SO or TCBZ-SO2 sub-lethal (15 μg/ml) (n = 6) or lethal (50 μg/ml) (n = 6) doses in DMSO (0.1%, v/v). Combining experiments undertaken after each abattoir visit, in total 60 *F. hepatica* were used for each TCBZ metabolite and concentration treatment, which were separated into six falcon tubes (ten *F. hepatica* per falcon tube) (n = 6). Viability was considered a score >1, reduced viability was considered a score of 1 and non-viable was considered a score of 0.

Supplementary data S2. The amount of anthelmintic metabolite (pg/particle^9^) found in EVs, determined by spectrum peak area quantification (ng) (Xcalibur software, Version 3.0) and qNano particle analysis sample concentration (particle/ml) in all anthelmintic exposure samples and control samples.

## Data Availability

All data generated and analysed during this study are included in this article

## References

[1] Charlier J, Vercruysse J, Morgan E, Van Dijk J, Williams D (2014). Recent advances in the diagnosis, impact on production and prediction of *Fasciola hepatica* in cattle. Parasitology.

[2] Omar MA, Metwally AM, Sultan K (2013). Molecular and phylogenetic status of *Fasciola* sp., of cattle in Qena, Upper Egypt. Pak. J. Biol. Sci..

[3] WHO. Fascioliasis. file:: https://www.who.int/foodborne_trematode_infections/fascioliasis/en/ (2015).

[4] Directorate-General for Research and Innovation (European Commision) A decade of EU-funded Animal Health. *EU publications* (2012).

[5] Brennan GP (2007). Understanding triclabendazole resistance. Exp. Mol. Pathol..

[6] Lipkowitz KB, McCracken RO (1991). A molecular modeling approach to in vivo efficacy of triclabendazole. J. Parasitol..

[7] Hennessy DR, Lacey E, Steel JW, Prichard RK (1987). The kinetics of triclabendazole disposition in sheep. J. Vet. Pharmacol. Ther..

[8] Virkel G, Lifschitz A, Sallovitz J, Pis A, Lanusse C (2006). Assessment of the main metabolism pathways for the flukicidal compound triclabendazole in sheep. J. Vet. Pharmacol. Ther..

[9] Halferty L, Brennan GP, Trudgett A, Hoey L, Fairweather I (2009). Relative activity of triclabendazole metabolites against the liver fluke, *Fasciola hepatica*. Vet. Parasitol..

[10] Stitt AW, Fairweather I (1994). The effect of the sulfoxide metabolite of triclabendazole (Fasinex) on the tegument of mature and immature stages of the liver fluke, *Fasciola hepatica*. Parasitology.

[11] Stitt AW, Fairweather I (1993). Fasciola hepatica: the effect of the microtubule inhibitors colchicine and tubulozole-C on the ultrastructure of the adult fluke. Parasitology.

[12] Robinson MW, Trudgett A, Hoey EM, Fairweather I (2002). Triclabendazole-resistant *Fasciola hepatica*: beta-tubulin and response to in vitro treatment with triclabendazole. Parasitology.

[13] Hanna REB, Scarcella S, Solana H, McConnell S, Fairweather I (2012). Early onset of changes to the reproductive system of *Fasciola hepatica* following in vivo treatment with triclabendazole. Vet. Parasitol..

[14] Stitt AW, Fairweather I (1996). *Fasciola hepatica*: disruption of the vitelline cells in vitro by the sulphoxide metabolite of triclabendazole. Parasitol. Res..

[15] McConville M (2006). Adult triclabendazole-resistant *Fasciola hepatica*: surface and subsurface tegumental responses to in vitro treatment with the sulphoxide metabolite of the experimental fasciolicide compound alpha. Parasitology.

[16] Carr AW, McCracken RO, Stillwell WH (1993). Uncoupling of rat liver mitochondrial oxidative phosphorylation by the fasciolicide triclabendazole and its sulfoxide and sulfone metabolites. J. Parasitol..

[17] Stitt AW, Fairweather I, Mackender RO (1995). The effect of triclabendazole (‘Fasinex’) on protein synthesis by the liver fluke, *Fasciola hepatica*. Int. J. Parasitol..

[18] Bennett JL, Köhler P (1987). *Fasciola hepatica*: action in vitro of triclabendazole on immature and adult stages. Exp. Parasitol..

[19] Alvarez LI (2005). Altered drug influx/efflux and enhanced metabolic activity in triclabendazole-resistant liver flukes. Parasitology.

[20] Mottier L, Moreno L, Alvarez L, Virkel G, Lanusse C (2004). Measurement of triclabendazole and its metabolites in liver flukes: method development and full validation. J. Pharm. Biomed. Anal..

[21] Cwiklinski K (2015). The extracellular vesicles of the helminth pathogen, *Fasciola hepatica* : biogenesis pathways and cargo molecules involved in parasite pathogenesis. Mol. Cell. Proteom.

[22] de la Torre-Escudero E, Bennett APS, Clarke A, Brennan GP, Robinson MW (2016). Extracellular vesicle biogenesis in helminths: More than one route to the surface?. Trends Parasitol..

[23] Mathivanan S, Ji H, Simpson RJ (2010). Exosomes: extracellular organelles important in intercellular communication. J. Proteom..

[24] Théry C (2018). Minimal information for studies of extracellular vesicles 2018 (MISEV2018): a position statement of the International Society for Extracellular Vesicles and update of the MISEV2014 guidelines. J. Extracell. Vesicles.

[25] Harischandra H, Yuan W, Loghry HJ, Zamanian M, Kimber MJ (2018). Profiling extracellular vesicle release by the filarial nematode *Brugia malayi* reveals sex-specific differences in cargo and a sensitivity to ivermectin. PLoS Negl. Trop. Dis..

[26] Azmi AS, Bao B, Sarkar FH (2013). Exosomes in cancer development, metastasis, and drug resistance: a comprehensive review. Cancer Metastasis Rev..

[27] Kulkarni HM, Swamy CVB, Jagannadham MV (2014). Molecular characterization and functional analysis of outer membrane vesicles from the Antarctic bacterium *Pseudomonas syringae* suggest a possible response to environmental conditions. J. Proteome Res..

[28] Bauwens A, Kunsmann L, Karch H, Mellmann A, Bielaszewska M (2017). Antibiotic-mediated modulations of outer membrane vesicles in enterohemorrhagic *Escherichia coli* O104:H4 and O157:H7. Antimicrob. Agents Chemother..

[29] Devos S (2015). The effect of imipenem and diffusible signaling factors on the secretion of outer membrane vesicles and associated Ax21 proteins in *Stenotrophomonas maltophilia*. Front. Microbiol..

[30] Morphew RM (2014). In vitro biomarker discovery in the parasitic flatworm *Fasciola hepatica* for monitoring chemotherapeutic treatment. EuPA Open Proteom..

[31] Chemale G (2010). Comparative proteomic analysis of triclabendazole response in the liver fluke *Fasciola hepatica*. J. Proteom. Res..

[32] Davis CN (2019). The importance of extracellular vesicle purification for downstream analysis: a comparison of differential centrifugation and size exclusion chromatography for helminth pathogens. PLoS Negl. Trop. Dis..

[33] Akpanika GA (2017). Polyphenols from *Allanblackia floribunda* seeds: Identification, quantification and antioxidant activity. Food Chem..

[34] Sánchez-López CM (2020). Diversity of extracellular vesicles from different developmental stages of *Fasciola hepatica*. Int. J. Parasitol..

[35] Raposo G, Stoorvogel W (2013). Extracellular vesicles: exosomes, microvesicles, and friends. J. Cell Biol..

[36] Aubertin K (2016). Massive release of extracellular vesicles from cancer cells after photodynamic treatment or chemotherapy. Sci. Rep..

[37] Shao H (2012). Protein typing of circulating microvesicles allows real-time monitoring of glioblastoma therapy. Nat. Med..

[38] Mitchell PJ (2009). Can urinary exosomes act as treatment response markers in prostate cancer?. J. Transl. Med..

[39] Lv L-H (2012). Anticancer drugs cause release of exosomes with heat shock proteins from human hepatocellular carcinoma cells that elicit effective natural killer cell antitumor responses in vitro. J. Biol. Chem..

[40] Chapuy B (2008). Intracellular ABC transporter A3 confers multidrug resistance in leukemia cells by lysosomal drug sequestration. Leukemia.

[41] Ciravolo V (2012). Potential role of HER2-overexpressing exosomes in countering trastuzumab-based therapy. J. Cell. Physiol..

[42] Corcoran C (2012). Docetaxel-resistance in prostate cancer: evaluating associated phenotypic changes and potential for resistance transfer via exosomes. PLoS ONE.

[43] Federici C (2014). Exosome release and low pH belong to a framework of resistance of human melanoma cells to cisplatin. PLoS ONE.

[44] Katano K (2004). Confocal microscopic analysis of the interaction between cisplatin and the copper transporter ATP7B in human ovarian carcinoma cells. Clin. Cancer Res..

[45] Safaei R (2005). Intracellular localization and trafficking of fluorescein-labeled cisplatin in human ovarian carcinoma cells intracellular localization and trafficking of fluorescein-labeled cisplatin in human ovarian carcinoma cells. Clin. cancer Res..

[46] Wang J (2014). Bone marrow stromal cell-derived exosomes as communicators in drug resistance in multiple myeloma cells. Blood.

[47] Yin J (2012). Secretion of annexin A3 from ovarian cancer cells and its association with platinum resistance in ovarian cancer patients. J. Cell. Mol. Med..

[48] Shedden K, Xie XT, Chandaroy P, Chang YT, Rosania GR (2003). Expulsion of small molecules in vesicles shed by cancer cells: association with gene expression and chemosensitivity profiles. Cancer Res..

[49] Johnsen KB (2014). A comprehensive overview of exosomes as drug delivery vehicles—endogenous nanocarriers for targeted cancer therapy. Biochim. Biophys. Acta Rev. Cancer.

[50] Brophy PM, MacKintosh N, Morphew RM (2012). Anthelmintic metabolism in parasitic helminths: proteomic insights. Parasitology.

[51] Robinson MW, Lawson J, Trudgett A, Hoey EM, Fairweather I (2004). The comparative metabolism of triclabendazole sulphoxide by triclabendazole-susceptible and triclabendazole-resistant *Fasciola hepatica*. Parasitol. Res..

[52] Jefferies JR, Campbell AM, Van Rossum AJ, Barrett J, Brophy PM (2001). Proteomic analysis of *Fasciola hepatica* excretory–secretory products. Proteomics.

[53] Marcilla A (2012). Extracellular vesicles from parasitic helminths contain specific excretory/secretory proteins and are internalized in intestinal host cells. PLoS ONE.

[54] de la Torre-Escudero E (2019). Surface molecules of extracellular vesicles secreted by the helminth pathogen *Fasciola hepatica* direct their internalisation by host cells. PLoS Negl. Trop. Dis..

[55] MacKintosh N (2011). Tools for Monitoring Drug Resistant *Fasciola hepatica* in Cattle and Sheep.

