# Supplementary material for: Evidence of sequestration of triclabendazole and associated metabolites by extracellular vesicles of Fasciola hepatica
Source: Sci Rep. 2020 Aug 10;10:13445. doi: 10.1038/s41598-020-69970-4 (PMC7418001; doi:10.1038/s41598-020-69970-4)
Supplement: Supplementary file 1 — Supplementary data S1. (a) F. hepatica viability scores and their description for the assessment of in vitro anthelmintic exposure55 and (b) viability of F. hepatica (n = 420) survival after 10 h of in vitro maintenance, where culture samples had been subjected to DMSO (0.1%, v/v) only (treatment control) (n = 6) or either TCBZ, TCBZ-SO or TCBZ-SO2 sub-lethal (15 μg/ml) (n = 6) or lethal (50 μg/ml) (n = 6) doses in DMSO (0.1%, v/v). Combining experiments undertaken after each abattoir visit, in total 60 F. hepatica were used for each TCBZ metabolite and concentration treatment, which were separated into six falcon tubes (ten F. hepatica per falcon tube) (n = 6). Viability was considered a score >1, reduced viability was considered a score of 1 and non-viable was considered a score of 0. [file 41598_2020_69970_MOESM1_ESM.docx]

Evidence of sequestration of triclabendazole and associated metabolites by extracellular vesicles of *Fasciola hepatica*

Chelsea N. Davis, Ana Winters, Ivana Milic, Andrew Devitt, Alan Cookson, Peter M. Brophy and Russell M. Morphew

a)

| Viability score | Description |
| --- | --- |
| 0 | A second score of 1 |
| 1 | No movement |
| 2 | Rarely moving or moving very slow |
| 3 | Moving with >2 second pauses |
| 4 | Moving with <2 second pauses |
| 5 | Moving continuously |

b)

| Viability score | Viability of *F. hepatica* after 10 hours of in vitro maintenance | | | | | | |
| --- | --- | --- | --- | --- | --- | --- | --- |
|  | Treatment control | Lethal TCBZ | Sub-lethal TCBZ | Lethal TCBZ-SO_2_ | Sub-lethal TCBZ-SO_2_ | Lethal TCBZ-SO | Sub-lethal TCBZ-SO |
| 0 | 0 | 0 | 0 | 0 | 0 | 0 | 0 |
| 1 | 0 | 2 | 1 | 4 | 1 | 1 | 1 |
| 2 | 60 | 58 | 59 | 56 | 59 | 59 | 59 |
| 3 | 0 | 0 | 0 | 0 | 0 | 0 | 0 |
| 4 | 0 | 0 | 0 | 0 | 0 | 0 | 0 |
| 5 | 0 | 0 | 0 | 0 | 0 | 0 | 0 |
